# Enantio- and Chemo-Selective HPLC Analysis of Silodosin on an Amylose-Based Chiral Stationary Phase

**DOI:** 10.3390/molecules30091966

**Published:** 2025-04-29

**Authors:** Daniele Sadutto, Francesca Romana Mammone, Giulia D’Ettorre, Leo Zanitti, Daniela De Orsi, Romina Alfonsi, Francesca Prestinaci, Roberto Cirilli

**Affiliations:** Centre for the Control and Evaluation of Medicines, Chemical Medicines Unit, Istituto Superiore di Sanità, Viale Regina Elena 299, 00161 Rome, Italy; daniele.sadutto@iss.it (D.S.); francescaromana.mammone@uniroma1.it (F.R.M.); giulia.dettore@iss.it (G.D.); leo.zanitti@iss.it (L.Z.); daniela.deorsi@iss.it (D.D.O.); romina.alfonsi@iss.it (R.A.); francesca.prestinaci@iss.it (F.P.)

**Keywords:** silodosin, impurities, enantiomer, Japanese Pharmacopoeia, enantioselective HPLC, method validation

## Abstract

A direct enantio- and chemo-selective high-performance liquid chromatographic method was developed for determining the enantiomeric impurity of the chiral active pharmaceutical ingredient silodosin. The simultaneous separation of enantiomers of silodosin and its main organic related substances listed in the Japanese Pharmacopoeia (JP) monograph for drug substance was achieved on Chiralpak AD-3 (250 mm × 4.6 mm, 3 μm) column under normal-phase isocratic conditions. The optimized conditions employed the mixture *n*-heptane-ethanol-diethylamine (70:30:0.1) (*v*/*v*/*v*) as a mobile phase and a temperature of 35 °C. The complete separation of the enantiomers of silodosin and its main impurities was obtained within 12 min. The chromatographic method has been validated according to the International Conference on Harmonization (ICH) guidelines and compared with the method reported in the JP monograph. The standard curve for silodosin exhibited linearity (R^2^ > 0.999) within the concentration range of 1.13–2500 µg mL^−1^. The Chiralpak AD-3 has demonstrated a remarkable level of efficiency, enabling the attainment of limits of quantitation for silodosin of 1.13 µg mL^−1^ (equivalent to 0.057% of a sample solution of 2 mg mL^−1^) and ranging from 0.48 µg mL^−1^ to 1.94 µg mL^−1^ for other impurities.

## 1. Introduction

In the domain of pharmaceuticals, stereoselective interactions are of paramount importance, as the presence of one or the other enantiomer of a chiral drug can elicit either beneficial or deleterious effects [1]. This phenomenon gained significant prominence following the revelation of thalidomide’s teratogenic consequences [2]. Thalidomide was a drug used to alleviate nausea in pregnant women when administered as a racemic mixture during the 1960s. Within a few years, a global incidence of phocomelia emerged, affecting approximately 10,000 infants worldwide. This event was driven by the action of the (*S*)-enantiomer, leading to a high mortality rate, with approximately 50% of affected infants perishing. Many of the survivors exhibited concomitant defects in addition to limb deficiencies. In the years following the thalidomide disaster, many countries tightened their drug approval regulations.

In 1992, the U.S. Food & Drug Administration (FDA) promulgated a policy regarding stereoisomeric drugs [3]. This policy stipulates that the development of the racemic forms of chiral drugs must be supported by pharmacological and toxicological characterization of the single enantiomers. A preponderance of substantiating evidence has been demonstrated to support this policy. This evidence, moreover, is not merely limited to the thalidomide tragedy. Scientific studies have demonstrated that racemates are associated with safety concerns and result in an increased prevalence of adverse drug reactions. In contrast, single enantiomers have been demonstrated to possess enhanced therapeutic properties and diminished toxicity. In consideration of the aforementioned findings, the pharmaceutical industry has adopted a novel marketing strategy for chiral drugs that entails an increased production and development of single-enantiomer forms of chiral drugs, as well as a substitution of previously approved racemates with their single-enantiomer versions, a practice referred to as “chiral switch” [4,5]. In the European Medicines Agency’s (EMA) report on medicines with new active ingredients scheduled for market release in 2023, more than 70% of the products listed are chiral, excluding biological medicines such as vaccines. Of the aforementioned 70%, more than two-thirds have been approved for use as single enantiomers [6]. A similar trend can be seen in approvals for the US market, where between 2010 and 2020 the FDA (Food and Drug Administration) approved mainly chiral drugs specifically marketed as single enantiomers (approximately 60% of the total) [7].

To address these challenges, chemists in the pharmaceutical industry are increasingly called upon to optimize, develop, and validate new analytical techniques capable of determining enantiomeric impurity in chiral active pharmaceutical ingredients (APIs). The development of these techniques is of paramount importance to ensure the effectiveness and/or safety of the treatment and must be carried out following current regulatory guidelines. High-performance liquid chromatography (HPLC) on the chiral stationary phase (CSP) constitutes a direct and efficacious enantioseparation technique [8] and is largely used for enantiomeric excess determinations in chiral compounds of pharmaceutical and synthetic interest. The popularity of enantioselective HPLC is contingent upon the accessibility of a wide range of commercial CSPs, which have demonstrated the ability to resolve a broad spectrum of chiral drug compounds through multimodal elution conditions, eliminating the need for prior chiral derivatization [9].

Among the enantiomerically pure chiral pharmaceuticals in current use, silodosin (SLD) is among the most prescribed and marketed drugs for the treatment of benign prostatic hyperplasia (BPH) and lower urinary tract symptoms (LUTS). The therapeutic efficacy of SLD can be attributed to the role of the (*R*) enantiomer as an antagonist of the α1A-adrenoreceptor (AR) family [10]. The (*S*) form is regarded as an impurity and necessitates the implementation of suitable analytical methodologies to monitor its presence and quantity in drug substances and the finished product.

To the best of our knowledge, there have been few enantioselective chromatographic methods proposed in the literature. Daicel Corporation has published two scientific notes [11,12] reporting the baseline separation between SLD and its enantiomeric impurity (*S*). The separation was performed using the amylose-base immobilized-type Chiralpak IF-3 and Chiralpak IG-3 columns in normal-phase and reversed-phase conditions, respectively. In a related study, Vali et al. [13] reported on the HPLC enantioseparation of SLD on the amylose-based coated–type Chiralpak AS CSP using a mobile phase consisting of *n*-hexane:ethanol:diethylamine (DEA) 60:40:0.01 (*v*/*v*/*v*). A capillary electrophoresis method has also been reported for the determination of chiral purity of SLD using carboxymethyl-β-cyclodextrin as a chiral selector [14].

The enantioselective methods documented in the literature for the enantioseparation of SLD have good enantioselectivity, but no evidence has been reported on the ability to separate the enantiomers from other potential impurities. This omission is not intentional, but it can be explained by two factors. Firstly, the cost of pharmaceutical impurities is often exorbitant. Secondly, impurities are sometimes difficult to source in the pharmaceutical marketplace.

**Figure 1:**
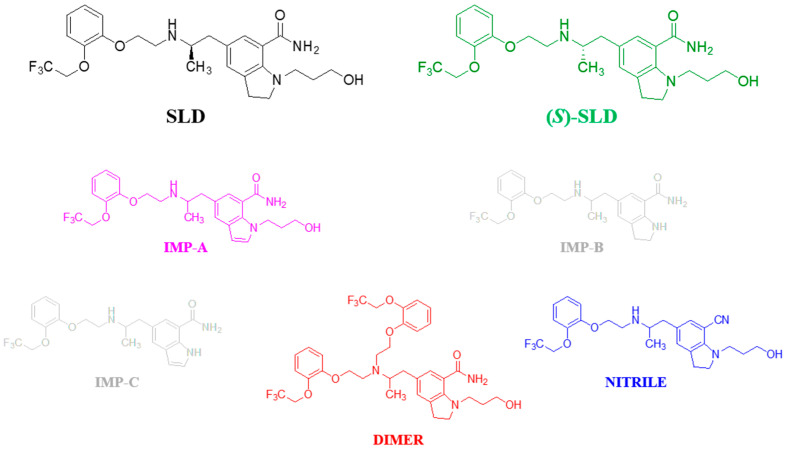
Chemical structures and abbreviations of silodosin (SLD) and its potential organic impurities. (*S*)-SLD: (*S*)-silodosin.

It is the responsibility of the regulatory agencies to establish conditions that satisfy the rigorous requirements of chemo-selectivity and enantio-selectivity for HPLC analysis [15,16,17,18]. In 2006, SLD received initial approval as a medicinal product in Japan. This approval was subsequently followed by the approval of SLD in both the United States (2008) and Europe (2009) [19]. Only in the Japanese Pharmacopoeia (JP) monograph (XVIII edition) for the API SLD is a normal-phase HPLC method that is able to separate SLD from its enantiomer (referred to (*S*)-SLD) is described [20]. The direct enantioselective separation is achieved on the CSP formed by cellulose tris (4-methylbenzoate) coated on 10 μm silica particles using a mobile phase consisting of *n*-hexane-ethanol-diethylamine 93:7:10 (*v*/*v*/*v*). Under these conditions, the flow rate is adjusted so that the retention time of SLD is about 29 min. In addition to the enantiomeric impurity (*S*)-SLD, the other impurities associated with the SLD drug substance listed in the JP monograph are primarily of synthetic origin and are impurities A, B, and C (here referred to as IMP-A, IMP-B, and IMP-C). (*S*)-SLD is expected to elute before (*R*)-SLD with a relative retention time (RRT) of 0.8. The structures of these substances are shown in Figure 1, along with the structures of two additional potential impurities, named DIMER and NITRILE.

In recent years, it has become evident that significant progress has been made in the production of more efficient chiral packing materials for HPLC enantioseparation [21]. The process of immobilizing the selector on the silica support and the miniaturization of the silica particle size have seen significant improvements. Specifically, the silica particle size has decreased from 10 μm, which was the standard in the 1980s, to the current sub-2 μm size [22]. Therefore, to enhance the reliability of analytical methods reported in Pharmacopoeia, it is imperative to consider the integration of technological advancements. A critical examination of conventional methods is crucial to identify opportunities for enhancement, resulting in more consistent, robust, user-friendly, and sustainable approaches.

The main aim of the present work is to develop and validate a novel, precise, simple and sensitive direct enantio-selective HPLC method capable of determining the enantiomeric excess of SLD in the presence of its potential impurities, providing an upgrade of the chromatographic conditions reported in the pertinent JP monograph.

## 2. Results and Discussion

### 2.1. HPLC Enantioseparation Under Normal-Phase Conditions

The first objective of this work was to develop an enantioselective HPLC method suitable for the enantiomeric separation of SLD. Screening studies were performed in the normal phase mode, using different mixtures of *n*-hexane:ethanol and *n*-heptane:ethanol, with 0.1% DEA added, and using different polysaccharide-derivative-based CSPs. The choice of DEA as the basic additive is an important factor in improving peak symmetry and maintaining the active pharmaceutical ingredient (API) and impurities as free bases. The choice to replace *n*-hexane with *n*-heptane, the traditional solvent used to prepare normal phase eluents, was based on the objective of providing a less toxic option for the separation. *N*-hexane has been demonstrated to be significantly more volatile, capable of inducing peripheral neuropathy, and exhibits greater neurotoxicity than *n*-heptane [23]. The most optimal outcomes in terms of resolution and analysis time were achieved with the Chiralpak AD-3 250 mm × 4.6 mm column. Chiralpak AD-3 is a commercially available column that is packed with 3 µm silica macroporous particles onto which amylose tris (3,5-dimethylphenylcarbamate) (ADMPC) has been physically coated [24,25].

Among the various chiral chromatographic selectors developed and applied for the resolution of chiral compounds, ADMPC is noteworthy for its particularly interesting properties in terms of enantioselectivity [26,27,28]. At present, commercially available ADMPC-based CSPs are classified into two distinct categories: coated-type and immobilized-type. This classification is based on the anchoring procedure of the polysaccharide onto silica matrices. The immobilization procedure offers recognized advantages, such as robustness in a wide range of mobile phase compositions and the ability to create enantioselective conditions in the presence of medium-polarity solvents (i.e., acetone, dichloromethane, ethyl acetate) that are incompatible with coated-type CSPs. However, the recognition ability of immobilized CSPs using the same mixtures of *n*-hexane-alcohol (ethanol or 2-propanol) is typically lower than that of coated versions [24].

This observation was substantiated in the resolution of SLD. The resolution factor values obtained with the immobilized Chiralpak IA-3 were consistently lower in all conditions investigated, even in the presence of cosolvents such as ethyl acetate or dichloromethane.

**Figure 2:**
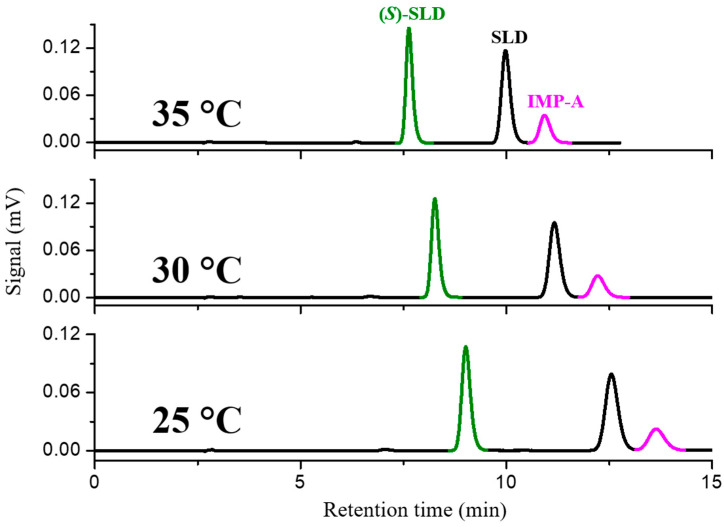
Temperature-variable HPLC chromatograms of SLD spiked with (*S*)-SLD and IMP-A. Chromatographic conditions: column, Chiralpak AD-3 (250 mm × 4.6 mm, 3 µm); mobile phase, *n*-heptane-ethanol-diethylamine 70:30:0.1 (*v*/*v*/*v*); temperature, as indicated in Figure 2; flow rate, 1.0 mL min^−1^; detection, UV at 270 nm.

The selectivity of Chiralpak AD-3 (250 mm × 4.6 mm, 3 µm) towards racemic SLD was optimized using a mobile phase comprising *n*-heptane-ethanol-DEA 70:30:0.1, at a flow rate of 1.0 mL min^−1^. At a column temperature of 25 °C the enantioseparation and resolution factors were 1.56 and 7.19, respectively. The chromatogram obtained in these conditions spiking the racemic mixture with impurity A, is reported in Figure 2.

In the development of an enantio-selective method for a chiral drug substance, meticulous attention must be taken to guarantee the capacity to discern not only the enantiomer impurity from API but also from other related substances.

The presence of co-eluting peaks related to these impurities can lead to misinterpretation of the enantiomer purity of the drug substance.

The separation of IMP-A from the enantiomers of SLD is expected to be a critical aspect of purity enantiomeric determination. This is attributable to the high structural similarity between the two chemical species. As illustrated in Figure 2, IMP-A does not interfere with the determination of enantiomeric purity because it does not coelute with (*S*)-SLD. Rather, it is eluted after SLD and separated with a resolution factor of 1.64.

It is important to remember that the quantitative determination of the (*S*) enantiomer is performed using a dilution reference standard solution of SLD [20].

Consequently, the complete selectivity of the method must be demonstrated in the context of its application for the simultaneous quantitative determination of all related substances.

In order to improve the separation of SLD/IMP-A, the temperature was progressively increased to 30 °C and 35 °C and the effect on the resolution of two critical pairs was evaluated. With increasing temperature, the enantioselectivity between SLD and its enantiomer decreased from 1.64 to 1.47. However, the resolution factor was still very high (i.e., Rs = 6.63). At 35 °C, the resolution between SLD and IMP-A increased to 2.13, which was the optimal condition to achieve the simultaneous enantio- and chemo-separation and to reduce the elution times.

**Figure 3:**
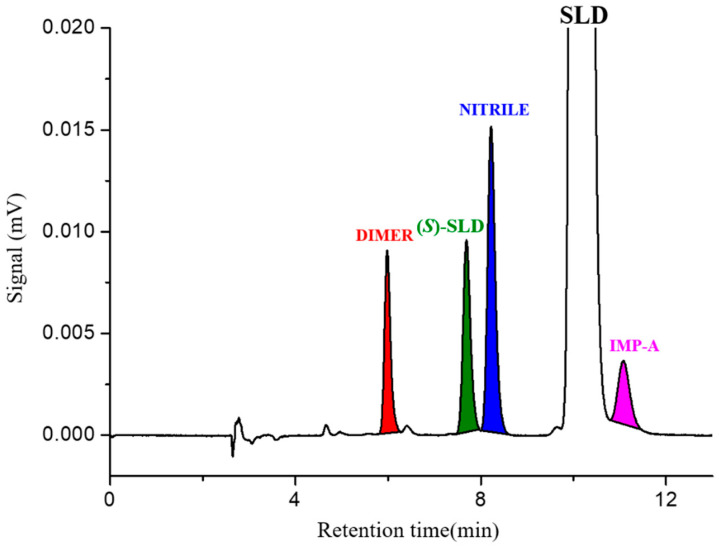
Typical HPLC chromatogram of SLD (conc. = 2.0 mg mL^−1^) spiked with 1.0% of (*S*)-SLD, IMP-A, NITRILE and DIMER. Chromatographic conditions: column, Chiralpak AD-3 (250 mm × 4.6 mm, 3 µm); mobile phase, *n*-heptane-ethanol-diethylamine 70:30:0.1 (*v*/*v*/*v*); temperature, 35 °C; flow rate, 1.0 mL min^−1^; detection, UV at 270 nm.

The unavailability of IMP-B and IMP-C does not allow us to demonstrate the selectivity of the conditions described above. It is worthy that both impurities differ from SLD by the absence of the hydroxypropyl chain, which is expected to be involved in the retention process [25]. It is therefore hypothesized that these impurities are retained less than (*S*)-SLD and do not interfere with the enantiomeric determination. The chromatogram corresponding to the analysis of a sample of SLD with a concentration of 2.0 mg mL^−1^ (sample solution) spiked with the remaining known impurities NITRILE and DIMER at 1.0 % is shown in Figure 3. The high selectivity of the chromatographic conditions used is highlighted by the resolution value recorded between the two critical peak pairs, SLD/IMP-A pair and (*S*)-SLD/NITRILE, which was greater than 1.5.

### 2.2. HPLC Enantioseparation Using the Method of the Japanese Pharmacopoeia Monograph

The JP monograph for the API SLD describes a normal phase HPLC method for the separation of SLD from (*S*)-SLD based on the use of a CSP formed by cellulose tris (4-methylbenzoate) coated on 10 μm silica particles [20]. The recommended mobile phase is a mixture of *n*-hexane-ethanol-diethylamine in a ratio of 93:7:10 (*v*/*v*/*v*). It should be noted that the concentration of diethylamine, and by extension, that of any other basic or acidic additive incorporated into the mobile phase, is allowed up to a maximum of 0.5–1.0% (*v*/*v*) [29] (specifications of the columns supplier). The use of higher concentrations of basic additives can lead to irreversible damage to the column. We assume that the reported percentage (i.e., 10%) is due to a typing error. The typical content of diethylamine used in the mobile phase is 0.1%, and such a percentage was used to mimic the enantioselective analysis.

**Figure 4:**
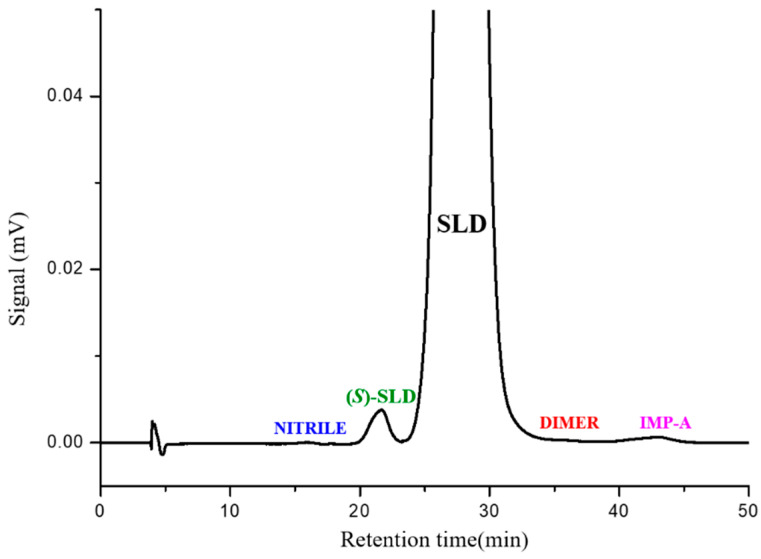
Typical HPLC chromatogram of SLD (conc. = 10.0 mg mL^−1^) spiked with (*S*)-SLD, IMP-A, NITRILE and DIMER obtained following the Japanese Pharmacopoeia method. Chromatographic conditions: column, Chiralcel OJ (250 mm × 4.6 mm, 10 µm); mobile phase, *n*-hexane-ethanol-diethylamine 93:7:0.1 (*v*/*v*/*v*).; temperature, 40 °C; flow rate, 0.75 mL min^−1^; detection, UV at 270 nm.

We decided to replicate the method described in the JP monograph. The result is shown in the chromatogram in Figure 4, which shows a typical chromatogram resulting from the HPLC analysis of SLD spiked with (*S*)-SLD, IMP-A, NITRILE, and DIMER impurities, carried out using the normal phase method described in the JP monograph for SLD drug substance. Accordingly, the sample solution was 10 mg mL^−1^ while the impurities were present at 0.5–1.0%. The column that was used was the Chiralcel OJ (250 mm × 4.6 mm, 10 µm), but other commercially available columns are equivalent and can be used as well.

As can be noted, with the flow rate set at 0.75 mL min^−1^ SLD was eluted at 28 min, (*S*)-SLD at 22 min, and IMP-A eluted at 42 min. The NITRILE impurity was the least retained species, and DIMER was eluted on the SLD peak tail. All impurities exhibited a clear separation from the enantiomeric peak, demonstrating resolution values greater than 1.5.

It is evident that certain features of the official method can be improved. The broad peaks observed in the analysis can be attributed to two factors: the low efficiency of the column packed with 10 µm particles, secondly, the delayed elution of the analytes. In order to address the aforementioned issues, it has been necessary to employ a sample solution with a very high concentration, specifically 10 mg mL^−1^, to obtain enantiomeric peaks of sufficient intensity. This approach ensures that the limit of quantification (LOQ) exceeds the signal-to-noise ratio (S/N) of 10 at a standard concentration fixed at 0.15%.

A further critical point pertains to the duration of the analysis. The apex of the broad peak pertinent to IMP-A is eluted at 42 min. Therefore, to ensure the complete elution of all potential impurities present in the standard or drug substance it is recommended to extend the analysis to a minimum of 50 min.

### 2.3. Method Validation Results

The proposed HPLC method, which is based on the Chiralpak AD-3 column, has been shown to be capable of separating a multi-component chemical mixture containing four related substances, including the (*S*)-enantiomer, from SLD within 12 min. Consequently, the optimized conditions identified have the potential for application and/or development not only for the determination of enantiomeric purity but also for the quantitative determination of other related substances investigated in this study.

Following the establishment of selectivity, the validation of the HPLC method was required to demonstrate that its characteristics (system suitability, precision, linearity accuracy, robustness and LOQ) are in accordance with ICH guidelines [30].

#### 2.3.1. System Suitability and Specificity

The system suitability solution was carried out at two levels (repeatability and specificity). The standard solution of SLD for system suitability was analyzed six times to evaluate the repeatability of the method. The obtained chromatograms are reported in Figure S1 of Supporting Materials. The percentage relative standard deviation (RSD%) of peak area and retention times were 0.63% and 0.02%, respectively (the proposed acceptance criterion is that the RSD% to be less than 2.0%), whereas the peak tailing was not more than 1.16 (the proposed acceptance criterion is that the peak tailing to be less than 1.5). The number of theoretical plates per meter (N/m) was about 37,000 (the proposed acceptance criterion is that the theoretical plates to be more than 25,000).

The system suitability tests have also been carried out also to establish the resolution between the enantiomers of SLD and the critical impurities IMP-A and NITRILE.

As shown in Table S1 the resolution values between the critical pairs were higher than the required limit of 1.5.

No interfering peak was observed in comparison with the blank and carry-over samples. The data obtained demonstrate that the method under validation is characterized by specificity.

#### 2.3.2. Linearity

The linearity of the HPLC protocol was assessed by analyzing separately solutions containing SLD and single impurities. The calibration curves obtained by linear regression analysis plotting the peak area against the concentration (mg mL^−1^) for SLD and impurities are presented in Table S1. A close examination of these data shows that all reported regression coefficients (R^2^) for SLD and its impurities are greater than 0.999. The correction factors (CFs) for each impurity reported in Table S1 were calculated by the response relative factors (RRFs) obtained by the following equation: RRF = slope of the linearity plot for the impurity/slope of the linearity plot for SLD. The CF is defined as the reciprocal of the RRF (CF = 1/RRF). Of particular note is the observation that IMP-A exhibits the lowest CF value, with a recorded value of 0.48. Consequently, the absorption capacity is reduced by approximately 50% in comparison to SLD (at a wavelength of 270 nm).

#### 2.3.3. Accuracy

Further analyses were conducted to assess the accuracy of the method through recovery tests. The recovery results are shown in Table S2. For all known impurities, recoveries ranged from 98% to 109%, including all different spiking tests performed at three different concentrations of 0.05, 0.30 and 1.00% relative to the silodosin test solution (2 mg mL^−1^). Satisfactory results were also obtained from the recovery test performed at the limit of quantification (LOQ) level (ranging from 101% to 107%).

#### 2.3.4. Precision and Repeatability

The precision of the method was determined by the repeatability (intra-day) and intermediate precision (inter-day) of recovery tests, which were conducted in triplicate (n = 3) (Table S2). About (*S*)-SLD, the method demonstrated a notable degree of intra-day repeatability, with an RSD% range of between ±0.04 and ±0.51. The RSD% range for the IMP-A, NITRILE and DIMER impurities was found to be satisfactory, falling between ±0.42 and ±3.31. The inter-day results, obtained by repeating the same recovery tests on three consecutive days and by another independent analyst, were included and expressed as RSD% between ±0.72 and ±8.23.

Finally, the precision of the system suitability was evaluated by analyzing the repeatability of injections. In this context, the retention time and peak areas of all impurities were observed in six replicate injections of 1.0% solution impurity mix and evaluated in terms of RSD%. In the case of DIMER impurity, the RSD% of the retention time and peak areas were 0.11% and 0.34%; for NITRILE impurity, 0.15% and 0.82%; IMP-A, 0.10% and 0.57%; (*S*)-SLD, 0.06% and 0.69%.

All precision and repeatability results obtained are within the limits of the established acceptance criteria.

#### 2.3.5. LOD and LOQ

The limit of detection (LOD) and LOQ concentrations are shown in Table S2. The results presented were selected based on S/N of 3 and 10, respectively. The most sensitive analytes were DIMER and NITRILE impurities with a LOQ of 0.48 µg mL^−1^ and 0.50 µg mL^−1^, respectively. SLD, (*S*)-SLD and IMP-A present LOQ values of 1.13, 1.13 and 1.94 µg mL^−1^, respectively.

#### 2.3.6. Robustness

A series of experiments was conducted to assess the impact on chromatographic separations of variations in the mobile phase and flow and column temperature. To this end, the system suitability solution was analyzed using *n*-heptane-ethanol-diethylamine 70:30:0.1 (*v*/*v*/*v*) solution with ± 1.0% of *n*-heptane and alcohol content, at flow rates of 0.9 and 1.1 mL min^−1^ and temperatures of 33 °C and 37 °C. The results obtained were within the acceptable limits. Indeed, the relative retention times of the known impurities were not significantly different from those of the reference conditions.

## 3. Materials and Methods

### 3.1. Chemical and Reagents

The properties (N° CAS, molecular mass, IUPAC name and % purity) of Silodosin and its impurities, including (*S*)-SLD (enantiomeric impurity), IMP-A, NITRILE and DIMER (see Figure 1) are reported in Table S3. Silodosin, NITRILE and DIMER impurities were kindly provided by Recordati S.p.A. IMP-A was purchased from LGC GMBH (Luckenwalde, Germany). Lastly, (*S*)-SLD was purchased from TLC Pharmaceutical Standards (Newmarket, Ontario, Canada). Stock and working solutions were prepared by appropriate dilution and mixing of the standards in ethanol, and they were subsequently stored in the dark at −20 °C. HPLC-grade solvents *n*-hexane, *n*-heptane and ethanol, and dimethylamine (99%) were supplied by Sigma-Aldrich (Milan, Italy).

Chromatographic analyses were carried out on the Chiralpak AD-3 (250 mm × 4.6 mm, 3 µm) and Chiralcel OJ (250 mm × 4.6 mm, 10 µm) columns distributed by Chiral Technologies Europe (Illkirch-Graffenstaden, France).

### 3.2. Instruments

HPLC analysis was performed on a Waters Alliance (Milford, MA, USA) e2695 system, which was coupled to a photodiode array detector (2998-PDA) and a column heater (Waters Alliance 30 cm). It operated on two separate channels with the mobile phases consisting of 0.1% DEA in *n*-heptane (mobile phase A) and 0.1% DEA in ethanol (mobile phase B). The injection volume was 5 µL and the detection wavelength was 270 nm. Subsequently, the data were acquired and processed using the Empower software (version 2.0).

### 3.3. Method Validation

The method validation was performed in accordance with the European Medicines Agency (EMA) ICH Q2 (R2) Guideline on the validation of analytical procedures [11], with minor adaptations. Specificity, linearity, accuracy, precision, sensitivity and robustness were evaluated.

#### 3.3.1. Preparation of System Suitability Solution

The system suitability solution containing only SLD was prepared by transferring 20.0 mg of SLD into a 10 mL volumetric flask and dissolving by ethanol to volume. The system suitability solution containing SLD and impurities was prepared by transferring 20.0 mg of SLD into a 10 mL volumetric flask followed by the addition of 5 mL of ethanol. Subsequently, 1 mL of impurities mix (with a concentration of 0.2 mg mL^−1^ for each one) was added. The resulting solution was maintained in an ultrasonic bath for 5 min and then diluted to volume with ethanol. Before the analysis, the solutions were filtered through a 0.20 µm nylon membrane filter.

#### 3.3.2. Specificity

Specificity was assessed in terms of selectivity. Firstly, the absence of interference peaks in the blank sample (ethanol) was evaluated and the potential carryover was assessed by injecting a blank sample after the highest calibration level. Subsequently, the system suitability solution was prepared for the system suitability test, which included SLD and its related substances. The parameter under observation was the resolution (Rs) between adjacent peaks, which was deemed acceptable if it exceeded the prescribed threshold of 1.5.

#### 3.3.3. Linearity

The linearity of the method was evaluated for each analyte by constructing a linear regression line, plotted as peak area versus concentration. The correlation coefficient (R^2^) was monitored to assess the linearity. To this end, calibration curves were prepared by dissolving the standards in ethanol. The SLD calibration curve was composed of nine points, with concentrations distributed across the expected range. The concentrations in the silodosin curve were 1.13, 2.25, 3.75, 25, 50, 500, 1000, 1500 and 2500 µg mL^−1^, while the seven-point concentrations of the impurities curve ranged from 0.48 µg mL^−1^ to 2.5 µg mL^−1^.

#### 3.3.4. Accuracy

The accuracy was determined by means of a recovery test, in which the SLD test solution (2 mg mL^−1^) was spiked with three different concentrations (0.05, 0.3, and 1.0%) for each impurity, in triplicate, in order to achieve a total of nine determinations. The lowest spiked level is equivalent to the LOQ. The accuracy of the method was evaluated based on the mean percent recovery of the known added amount of the analyte in the sample. A recovery range of 80% to 120% was deemed acceptable.

#### 3.3.5. Precision

The precision of the method was determined by measuring the repeatability (intra-day) and intermediate precision (inter-day). The parameter monitored to evaluate the precision of the method was the relative standard deviation (RSD%), which offers a measure for quantifying the variability in the outcomes obtained. The intra-day was evaluated on the basis of the recovery results obtained on the same day, with triplicate measurements taken for each spiked level. An additional analyst used a three-day period to perform the inter-day variability analysis, repeating the 0.3% and 1.0% recovery tests. Furthermore, the precision of the system suitability solution was determined by measuring the repeatability of the retention time and peak areas of silodosin and each impurity on replicate injections (n = 6). The results were reported as RSD%.

#### 3.3.6. Sensitivity

The sensitivity was determined by evaluating the LOD and LOQ parameters. The LOD and LOQ values of silodosin and related impurities were determined by injecting a series of diluted solutions, where the lowest concentration was selected on the basis of an equal signal-to-noise ratio (S/N) of 3 and 10, respectively, in accordance with the EP guidelines.

#### 3.3.7. Robustness

Finally, the robustness of the analytical procedure was evaluated to ascertain its reliability in response to deliberate variations in procedure parameters, including minor alterations in the percentage of the mobile phase, in flow rate and the temperature of the column temperature.

## 4. Conclusions

A novel, simple, sensitive, specific, reproducible and linear isocratic normal-phase HPLC protocol based on the Chiralpak AD-3 (250 mm × 4.6 mm, 3.0 µm) column was developed and validated for the separation of SLD and (*S*)-SLD in a single chromatographic run without suffering from interference by other three potential impurities. The modifications implemented in the JPN SLD monograph have provided the following improvements: (i) faster elution times for all substances present in the mixed sample, resulting in a gain in analysis time and solvent consumption; (ii) substitution of *n*-hexane by less toxic *n*-heptane in the mobile phase; (iii) precise composition of the mobile phase using the correct concentration of basic additive compatible with the polysaccharide-bases CSPs; (iv) reduced SLD consumption; when preparing the sample solution, 2 mg of SLD was used for a single analysis instead of 10 mg. It follows that the adoption of the proposed analytical approach holds significant potential for routine application in quality control testing to assess enantiomeric purity in SLD-containing working standards and drug substances.

## Data Availability

Data are contained within the article and Supplementary Materials.

## Supplementary Materials

The following supporting information can be downloaded at: https://www.mdpi.com/article/10.3390/molecules30091966/s1, Figure S1: Repeatability (n=6) chromatograms of the silodosin test solution (2 mg mL^−1^); Table S1: Retention time, calibration curve equation, regression coefficient (R^2^), resolution factor (Rs) and Correction Factor (CF) of silodosin and its impurities; Table S2: Silodosin and its chiral impurities: limit of detection (LOD), limit of quantification (LOQ), recovery (%) with their relative standard deviations (±RSD); Table S3: IUPAC name, molecular formula, number CAS, molecular weight (MW]) and purity percentage of silodosin and its impurities.
