# Supplementary material for: Enantio- and Chemo-Selective HPLC Analysis of Silodosin on an Amylose-Based Chiral Stationary Phase
Source: Molecules. 2025 Apr 29;30(9):1966. doi: 10.3390/molecules30091966 (PMC12073136; doi:10.3390/molecules30091966)
Supplement: Supplementary file 1 [file molecules-30-01966-s001.zip › molecules-3557860-supplementary.pdf]

Supplementary materials

Enantio- and chemo-selective HPLC analysis of silodosin on an amylose-based chiral stationary phase

Daniele Sadutto[1], Francesca Romana Mammone[1], Giulia D'Ettorre[1], Leo Zanitti[1], Daniela De Orsi[1], Romina Alfonsi[1], Francesca Prestinaci[1] and Roberto Cirilli[1,*]

1 Centre for the Control and Evaluation of Medicines, Chemical Medicines Unit, Istituto Superiore di Sanità, Viale Regina Elena 299, 00161 Rome, Italy.

*Correspondence: roberto.cirilli@iss.it

Figure S1. Repeatability (n=6) chromatograms of the silodosin test solution (2 mg mL^{-1}).

Chromatographic conditions: Column, Chiralpak AD-3 (25 0mm x 4.6 mm, 3µm), mobile phase, n-heptane-ethanol-diethylamine 70:30:0.1 (v/v/v); temperature, 35°C; flow 1.0 mL min^{-1}; detention, UV at 270 nm.

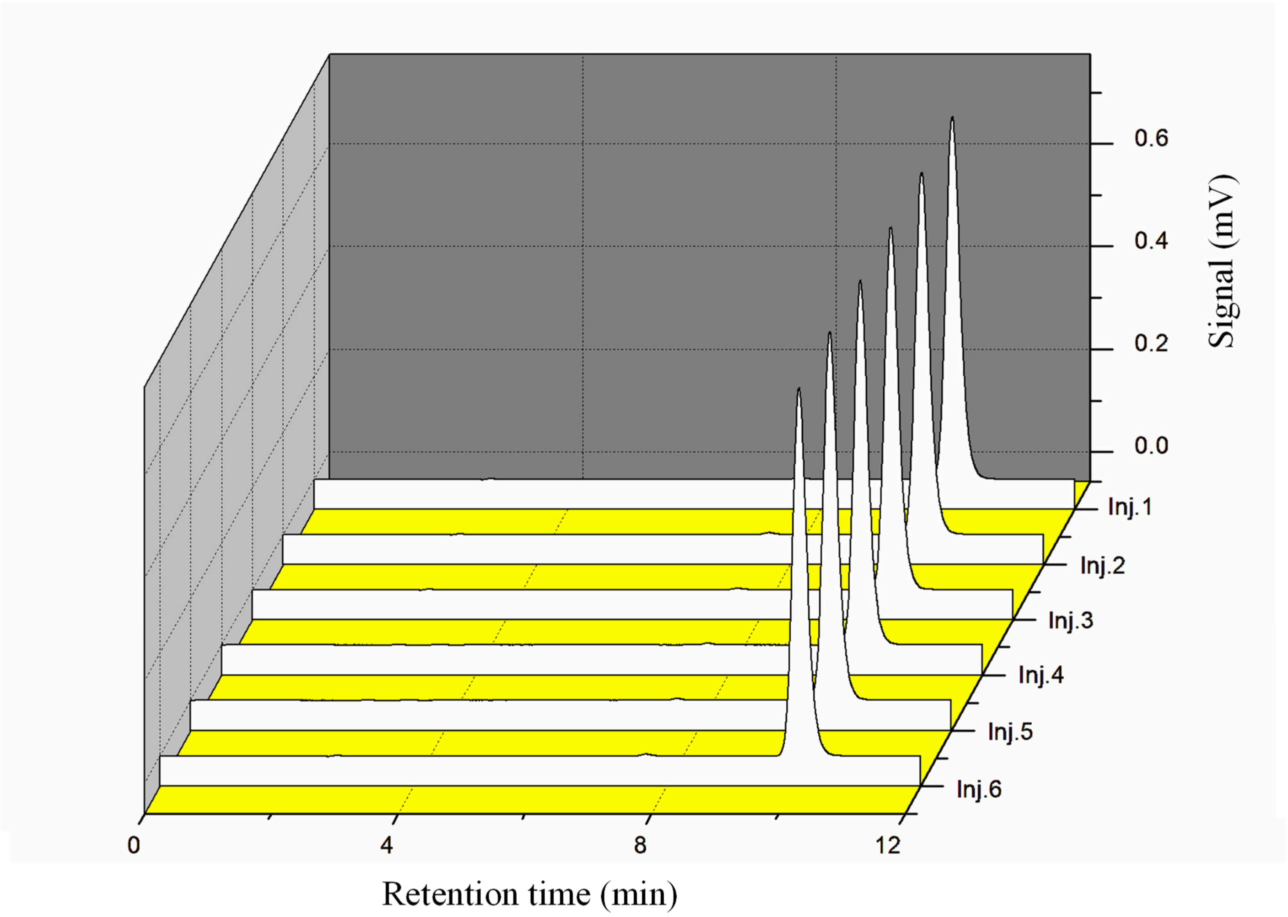

Table S1. Retention time, calibration curve equation, regression coefficient (R^2), resolution factor (Rs) and Correction Factor (CF) of silodosin and its impurities.

Compound	Retention time (min)	Calibration curve equation	R^2	Rs	CF
DIMER	6.00	y = 2.61×10−7x – 0.00010	0.999	-	0.84
(*S*)-SLD	7.71	y = 2.10×10−7x – 0.00140	0.999	6.63	1.00
NITRILE	8.25	y = 1.69×10−7x – 0.00005	0.999	1.90	1.29
SLD	10.17	y = 2.18×10−7x – 0.00140	0.999	5.50	-
IMP-A	11.13	y = 4.53×10−7x – 0.00070	0.999	2.13	0.48

Table S2. Silodosin and its chiral impurities: limit of detection (LOD), limit of quantification (LOQ), recovery (%) with their relative standard deviations (±RSD).

Compound	LOD (µg/mL)	LOQ (µg/mL)	% Recovery (%) (±RSD%) (n = 3) intra-day			% Recovery (%) (±RSD%) (n =3) inter-day	
			**0.05%**	**0.3%**	**1%**	**0.3%**	**1%**
SLD	0.34	1.13	/	/	/	/	/
(*S*)-SLD	0.34	1.13	101 (±0.51)	108 (±0.45)	100 (±0.04)	109 (±1.73)	100 (±0.72)
IMP-A	0.59	1.94	107 (±0.56)	104 (±3.31)	98 (±2.41)	108 (±3.04)	100 (±1.92)
NITRILE	0.15	0.50	101 (±1.57)	109 (±2.55)	105 (±2.29)	116 (±3.36)	105 (±2.16)
DIMER	0.14	0.48	100 (±1.73)	103 (± 0.69)	105 (±0.42)	113 (±8.23)	108 (±2.69)

Table S3. IUPAC name, molecular formula, number CAS, molecular weight (MW]) and purity percentage of silodosin and its impurities.

Compound	IUPAC name	Molecular Formula	N° CAS	MW	Purity %
SLD	1-(3-hydroxypropyl)-5-[(2R)-2-[2-[2-(2,2,2-trifluoroethoxy)phenoxy]ethylamino]propyl]-2,3-dihydroindole-7-carboxamide	$C_{25}H_{32}F_3N_3O_4$	160970-54-7	495.5	100.0
(*S*)-SLD	1-(3-hydroxypropyl)-5-[(2S)-2-[2-[2-(2,2,2-trifluoroethoxy)phenoxy]ethylamino]propyl]-2,3-dihydroindole-7-carboxamide	$C_{25}H_{32}F_3N_3O_4$	2182279-45-2	495.5	96.0
IMP-A	1-(3-hydroxypropyl)-5-[(2R)-2-[2-[2-(2,2,2-trifluoroethoxy)phenoxy]ethylamino]propyl]indole-7-carboxamide	$C_{25}H_{30}F_3N_3O_4$	175870-21-0	493.5	97.0
NITRILE	1-(3-hydroxypropyl)-5-[(2R)-2-[2-[2-(2,2,2-trifluoroethoxy)phenoxy]ethylamino]propyl]-2,3-dihydroindole-7-carbonitrile	$C_{25}H_{30}F_3N_3O_3$	885340-13-6	477.5	98.8
DIMER	5-[(2R)-2-[Bis[2-[2-(2,2,2-trifluoroethoxy)phenoxy]ethyl]amino]propyl]-2,3-dihydro-1-(3-hydroxypropyl)-1H-indole-7-carboxamide	$C_{35}H_{41}F_6N_3O_6$	1453221-45-8	713.7	95.2
